# Volume isn't openness: misaligned assessment and Open Science adoption in Ecuador

**DOI:** 10.3389/frma.2025.1707881

**Published:** 2026-01-14

**Authors:** Julio Guerra, Miguel Naranjo-Toro, Andrea Basantes-Andrade

**Affiliations:** Universidad Técnica del Norte, Ibarra, Ecuador

**Keywords:** Ecuador, open access, Open Science, research assessment, science policy

## Abstract

Open Science aims to make research more transparent, reusable, and socially valuable, yet adoption may lag where assessment emphasizes journal prestige over openness. This study examines how research-assessment incentives align with Open Science practices in Ecuador and identifies policy levers associated with change. Using a mixed-methods design, we combine a review of national and institutional policies, a bibliometric analysis of Ecuador-affiliated outputs from 2013–2023, and a nationwide researcher survey (*n* ≈ 418), analyzed with multilevel logistic models, multinomial logit, and negative binomial regressions. Scientific output increased markedly, peaking at 5,070 articles in 2023; 66.7% were open access, predominantly via gold routes. In 2021, 59.3% of citations were self-citations. Despite high familiarity with Open Science (85%), implementation was limited: 22% reported depositing data and 35% publishing via diamond or gold routes. Greater reliance on journal-centric metrics was associated with lower odds of adopting open practices (odds ratio ≈ 0.72), while comprehensive institutional support—repositories with deposit mandates, research-data services, and licensing guidance—was associated with higher odds (odds ratio ≈ 1.65). Sensitivity to article processing charges was associated with shifts toward green and diamond routes. Findings suggest that socio-institutional factors dominate barriers and that aligning rules, services, and responsible assessment may help make openness the default, improving quality, equity, and reuse.

## 1 Introduction

Open Science (OS) has consolidated as an international framework that promotes transparency, accessibility, and collaboration across the entire research cycle. The UNESCO Recommendation on Open Science defines it as a set of values, principles, and practices aimed at reducing technological and knowledge gaps, strengthening quality and reproducibility, and connecting science with societal needs (UNESCO, 2021). In parallel, the European Union has embedded OS as a pillar of Horizon Europe, with mandatory open access (OA) to publications, data opening “as open as possible, as closed as necessary,” and guidance on open evaluation and stakeholder engagement (Arentoft et al., 2022). These policies accelerate the transition toward openness, while quantitative evidence confirms the sustained global growth of open access (Piwowar et al., 2018; Vicente-Saez and Martínez-Fuentes, 2018).

Despite this momentum, OS adoption is uneven and entails equity risks, especially across the Global South. Article processing charges operate as an economic barrier that stratifies scholarly communication and penalizes systems with lower purchasing power (Klebel and Ross-Hellauer, 2023). Moreover, expanding open practices without appropriate recognition and assessment frameworks can amplify existing asymmetries (Ross-Hellauer et al., 2022). In Latin America, “diamond OA” initiatives such as Redalyc/AmeliCA provide a noncommercial, cooperative model that sustains openness without APCs, illustrating strategies better suited to resource-constrained contexts (Aguado-López et al., 2018; Chiarelli et al., 2024; UNESCO, 2024).

Beyond distributional concerns, recent evidence indicates a sharp escalation and opacity in article processing charges (APCs), with global APC expenditures nearly tripling between 2019 and 2023 and hybrid fees outpacing gold fees (Haustein et al., 2024). Library-side cost analyses likewise document upward pressure and weak price signals in APC markets (Borrego, 2024). From a Global South perspective, APC-based models can function as a structural barrier, producing epistemic and testimonial injustice when authors' ability to publish is conditioned on ability to pay (Cox, 2023). These dynamics reinforce our premise: without responsible assessment and publicly supported open infrastructures, openness risks reproducing inequalities rather than reducing them.

In Ecuador, scientific output has increased notably over the last decade and now reaches thousands of documents annually in Scopus, reflecting expansion of the R&D system and its internationalization (SCImago Journal & Country Rank, 2025). This surge unfolds within a regulatory architecture in which the Organic Law of Higher Education (LOES) enshrines the dissemination and application of knowledge as a core function of the system (Asamblea Nacional del Ecuador [National Assembly of Ecuador], 2010; Asamblea Nacional del Ecuador, 2018), and in which the Council for Quality Assurance in Higher Education (Consejo de Aseguramiento de la Calidad de la Educación Superior, CACES) has established criteria and processes for the evaluation and validation of scholarly journals, promoting editorial and indexation standards [Consejo de Aseguramiento de la Calidad de la Educación Superior (CACES) [Ecuadorian Council for Quality Assurance in Higher Education], 2019].

However, a misalignment persists between assessment incentives and OS principles. In practice, academic progression and institutional accreditation are anchored in metrics and indexation logics (e.g., presence in WoS/Scopus and journal impact factors), which incentivize journal-centric prestige over open practices such as preprints, data sharing, institutional repository deposit, or diamond publishing. This mismatch has been recognized internationally, giving rise to initiatives such as the Agreement on Reforming Research Assessment (CoARA), which promote valuing the quality and diversity of outputs over single-number metrics (Arentoft et al., 2022). Likewise, phenomena such as strategic self-citation and the prioritization of bibliometric indicators over open exchange of inputs are documented and suggest unintended effects of prevailing assessment regimes (Ioannidis et al., 2019; Baccini et al., 2019).

This article contends that, in the Ecuadorian case, the principal barriers to full OS adoption are not technological but sociocultural and institutional: the lack of tangible rewards for open practices, perceived intellectual-property risks, and weak organizational support for infrastructures and services. To examine this claim, it employs a mixed-methods study that combines policy review, bibliometric analysis, and a researcher survey with three objectives: (i) to analyze attitudes and practices toward OS; (ii) to map incentives and disincentives embedded in the national assessment system; and (iii) to propose institutional adjustments—recognition of data and preprints, support for repositories and diamond journals, and alignment with international frameworks—that make the transition to openness sustainable. In doing so, it contributes applied evidence for context-sensitive OS implementation in developing countries, shifting the focus from purely technical fixes to cultural change and assessment reform.

## 2 Theoretical framework

The transition toward Open Science (OS) entails an institutional shift in the norms, practices, and incentives that govern scientific activity. To study barriers and levers in Ecuador, the analysis is structured around three analytical frameworks: (i) Open Science principles and components; (ii) institutional theory and path dependence; and (iii) the sociology of research assessment and responsible metrics. This multilevel approach explains cultural and organizational resistance that persists even when technical capacities are available (UNESCO, 2021; European Commission, 2022; Hicks et al., 2015).

### 2.1 Open Science

The UNESCO Recommendation on European Commission (2021) defines OS as an international normative instrument that promotes scientific knowledge that is open, accessible, and reusable, and it identifies four pillars: open scientific knowledge (publications, data, software, educational resources), open infrastructures, open scientific communication, and social engagement in co-creation (UNESCO, 2021). In the European Union, Horizon Europe (2021–2027) embeds OS practices in criteria for excellence and implementation, with mandatory open access to publications, data-management plans, and the principle “as open as possible, as closed as necessary” (European Commission, 2022). Empirical evidence also shows that both the share of open-access literature and its relative impact have grown steadily (Piwowar et al., 2018; Vicente-Saez and Martínez-Fuentes, 2021).

This framework challenges the traditional “social contract” of science centered on closed publication and priority of discovery by valuing process transparency (data, methods, code), collaboration, and social relevance as dimensions of scientific quality (UNESCO, 2021; European Commission, 2022). Reform initiatives—such as DORA (2012), the Leiden Manifesto (Hicks et al., 2015), and the Agreement on Reforming Research Assessment [Coalition for Advancing Research Assessment (CoARA), 2022]—propose shifting emphasis away from single-number metrics toward qualitative judgment, diversity of outputs, and recognition of open practices [DORA, 2012; Hicks et al., 2015; Coalition for Advancing Research Assessment (CoARA), 2022].

### 2.2 Institutional theory and path dependence

From institutional theory, institutions are the “rules of the game” (formal and informal) that structure human interaction; they generate stability but also path dependence that is associated with lock-in when existing incentives discourage change (North, 1990). In higher education, regulations, assessment models, and cultural norms of scientific “success” form an arrangement that can align with or clash against OS. In Ecuador, LOES—with current reforms—enshrines knowledge dissemination as a core function, and CACES sets criteria and guidelines for institutional evaluation and journal validation; however, the operationalization of these frameworks can anchor incentives in indexation and journal-centric metrics, hindering the uptake of open practices if they are not explicitly recognized and rewarded [Asamblea Nacional del Ecuador [National Assembly of Ecuador], 2010; Asamblea Nacional del Ecuador, 2018, 2022; Consejo de Aseguramiento de la Calidad de la Educación Superior (CACES) [Ecuadorian Council for Quality Assurance in Higher Education], 2019; Consejo de Aseguramiento de la Calidad de la Educación Superior (CACES), 2023; North, 1990].

At the level of informal norms, the scientific ethos (Merton, 1973) historically upholds communalism, universalism, disinterestedness, and organized skepticism; nonetheless, in practice, incentive regimes may reconfigure behaviors toward prestige goals (e.g., JIF, quartiles) rather than toward openness and reproducibility. Path dependence helps explain why, even under pro-OS rhetoric, academies and organizations maintain inherited routines and metrics (Merton, 1973; North, 1990).

### 2.3 Research-assessment metrics

The sociology of evaluation warns that metrics are not neutral: they shape behavior and can create perverse incentives if used simplistically. The Leiden Manifesto (Hicks et al., 2015) and The Metric Tide (Wilsdon et al., 2015) recommend responsible measurement: combining qualitative assessment with contextualized indicators, avoiding the use of JIF to judge articles or people, and recognizing a variety of contributions (data, software, public engagement, inter-/transdisciplinary collaboration). In the same vein, DORA and CoARA call for explicit criteria that value OS practices (e.g., data plans, repository deposit, preprints, open peer review) as part of scientific merit [DORA, 2012; European Commission, 2022; Coalition for Advancing Research Assessment (CoARA), 2022]. Under these frameworks, resistance to OS should not be read as normative rejection but as a rational response to assessment systems in which rewards concentrate on specific indicators; hence the need to align incentives with practices of openness, transparency, and reuse (Hicks et al., 2015; Wilsdon et al., 2015; UNESCO, 2021).

### 2.4 Comparative regulatory frameworks in South America

Latin America offers an alternative pathway anchored in community-owned infrastructures—national and regional repositories (e.g., LA Referencia) and diamond OA platforms (e.g., Redalyc/AmeliCA)—that prioritize no-fee publishing and metadata interoperability as public goods (LA Referencia, 2022; Becerril, 2022). Recent international policy fora have converged on sustaining diamond OA through shared services, persistent identifiers, and funding mechanisms coordinated across regions (Science Europe et al., 2022).

To contextualize the Ecuadorian case, regional OS-promoting frameworks are summarized:

- Argentina: Law 26.899 (2013) creates the National System of Digital Repositories and mandates open-access deposit of results funded with public resources (Ministry of Science/Official Gazette; Argentina, 2013; Resolución 753/2016).
- Peru: CONCYTEC issues the ALICIA National Repository Directive (2016, 2020, 2021, 2022), setting guidelines, interoperability, and deposit obligations [Consejo Nacional de Ciencia, Tecnología e Innovación Tecnológica (CONCYTEC), 2016, 2020, 2021, 2022].
- Colombia: The National Open Science Policy (2022–2031) articulates open access and open data through the Colombian Scientific Information Network [Ministerio de Ciencia, Tecnología e Innovación (MinCiencias, Colombia), 2022].
- Chile: ANID adopts the Open-Access Policy for Scientific Information and Research Data funded with Gobierno de Ecuador - Secretaría de Gobierno Abierto (2022), including deposit and data-management obligations [Agencia Nacional de Investigación y Desarrollo (ANID), 2022, 2023].
- Brazil: Agencies and national networks advance via institutional policies (e.g., FAPESP Open Science, SciELO, and OA guidelines), emphasizing equity and sustainability [FAPESP (Fundação de Amparo à Pesquisa do Estado de São Paulo), n.d.; Reis-Santos and Braga, 2022].

In Ecuador, beyond LOES and its reforms, CACES publishes methodological guides for journal evaluation/validation and models for external institutional evaluation [Consejo de Aseguramiento de la Calidad de la Educación Superior (CACES) [Ecuadorian Council for Quality Assurance in Higher Education], 2019; Consejo de Aseguramiento de la Calidad de la Educación Superior (CACES), 2023], which are key to aligning quality assurance with OS practices [Asamblea Nacional del Ecuador [National Assembly of Ecuador], 2010; Asamblea Nacional del Ecuador, 2018, 2022; Consejo de Aseguramiento de la Calidad de la Educación Superior (CACES) [Ecuadorian Council for Quality Assurance in Higher Education], 2019; Consejo de Aseguramiento de la Calidad de la Educación Superior (CACES), 2023].

## 3 Objectives and hypotheses

Consistent with the theoretical framework (OS as public policy and institutional change) and international recommendations on responsible assessment [UNESCO, 2021; DORA, 2012; Hicks et al., 2015; Coalition for Advancing Research Assessment (CoARA), 2022], the study sets one general objective and a set of specific objectives operationalizable via documentary analysis, bibliometrics, and a survey. These objectives translate into testable hypotheses that connect incentives, institutional capacities, and open practices (Piwowar et al., 2018; Wilsdon et al., 2015; Klebel and Ross-Hellauer, 2023).

### 3.1 General objective

Analyze in Ecuador the degree of alignment between assessment incentives and the adoption of OS practices, integrating regulatory review (LOES, CACES), bibliometric evidence, and a researcher survey, to propose institutional adjustments that enable a sustainable transition toward OS.

### 3.2 Specific objectives

- O1: Map the regulatory and quality-assurance architecture (LOES, regulations, CACES guides and related guidelines) and identify its convergence with international standards of responsible assessment.
- O2: Quantify openness in Ecuador-affiliated scientific output (OA routes: gold, green, hybrid, bronze; use of preprints; availability of data/code) and its evolution, using bibliographic and open sources.
- O3: Characterize OS attitudes, knowledge, and practices in the research community (data sharing, repository deposit, OA publishing, preprints) and perceived barriers (APC costs, IP risks, time, recognition).
- O4: Model determinants of OS adoption (e.g., assessment incentives, institutional support, disciplinary culture, international collaboration, OS literacy) via multivariate analysis of the survey and triangulation with bibliometric signals.
- O5: Propose policy and university-management recommendations (repository mandates and services; recognition of data/software/preprints in promotion; diamond-OA-type incentives) aligned with UNESCO, DORA, and CoARA.

### 3.3 Hypotheses

- H1 (Assessment incentives): Greater perceived emphasis on journal-centric metrics (e.g., JIF, WoS/Scopus quartiles) is associated with a lower probability of adopting OS practices (preprints, open data/code, repository deposit), controlling for field and seniority. Expected: negative coefficient [Hicks et al., 2015; Wilsdon et al., 2015; DORA, 2012; Coalition for Advancing Research Assessment (CoARA), 2022].
- H2 (Institutional support): The existence of an institutional repository, deposit mandates, and support services (data management, legal advisory) is positively associated with OS adoption. Expected: positive coefficient (Piwowar et al., 2018; UNESCO, 2021).
- H3 (APC costs): Publication costs (APCs) are negatively associated with gold/hybrid OA publishing and positively associated with preference for no-APC routes (green/diamond). Expected: negative for paid OA; positive for green/diamond (Klebel and Ross-Hellauer, 2023; Aguado-López et al., 2018).
- H4 (OS literacy): Greater knowledge of OS principles/tools [data-management plans, licenses, FAIR (Findable, Accessible, Interoperable, Reusable)] is positively associated with effective adoption. Expected: positive coefficient (Fecher and Friesike, 2014; Vicente-Saez and Martínez-Fuentes, 2018).
- H5 (IP risk): Higher perceived intellectual-property risk and patent prioritization are negatively associated with data/code sharing and open deposit. Expected: negative coefficient (Borgman, 2012, 2015; Tenopir et al., 2020).
- H6 (International collaboration): Collaboration with institutions in countries with OA mandates is positively associated with OA publishing, preprint use, and data sharing, via norm transfer. Expected: positive coefficient (Piwowar et al., 2018; Huang et al., 2020).
- H7 (Disciplinary culture): Field differences exist (e.g., greater propensity for preprints in STEM and for open data in the life sciences) that modulate OS adoption. Expected: significant fixed/random effects by discipline (Fraser et al., 2021; Larivière et al., 2015).
- H8 (Metric pressure and self-citation): Greater pressure from bibliometric indicators is associated with higher author-level and/or institutional self-citation rates, net of age and field. Expected: positive coefficient (Ioannidis et al., 2019; Fong and Wilhite, 2017).

## 4 Methodology

### 4.1 Study design

The study adopts an explanatory sequential mixed-methods design in three layers: (i) a normative–documentary analysis (frameworks and policies); (ii) a bibliometric analysis (output and openness); and (iii) a researcher survey to model attitudes, practices, and incentives. It integrates strands through convergent triangulation and meta-inferences addressing hypotheses H1–H3. For transparency, the protocol and materials (instruments, scripts, anonymized data) will be pre-registered on OSF prior to fieldwork and released in an open repository with a DOI [OSF (Open Science Framework), 2025].

### 4.2 Normative–documentary component

The study draws on the following sources as the normative and documentary base:

- International OS framework: UNESCO Recommendation on Open Science (UNESCO, 2021).
- European framework: “as open as possible, as closed as necessary” [European Commission, 2022; (Open Science) IPR Helpdesk (European IP Helpdesk), n.d.].
- Ecuadorian framework: LOES, the Higher Education Academic Regulations (CES), and CACES standards for evaluation/accreditation [Consejo de Educación Superior (CES), 2022; Consejo de Aseguramiento de la Calidad de la Educación Superior (CACES), 2023].
- South American comparators: Argentina (Law 26.899/2013), Peru (Law 30035/2013 and ALICIA), Chile [Agencia Nacional de Investigación y Desarrollo (ANID), 2022], and recent overviews in Brazil [Argentina, 2013; Perú (Ley No. 30035), 2013; Agencia Nacional de Investigación y Desarrollo (ANID), 2022; UnB/IBICT, 2025].

We perform a systematic reading and extraction of key provisions (definitions; deposit obligations; licenses; incentives and sanctions).

### 4.3 Bibliometric component

The bibliometric component employs OpenAlex (stable snapshot; reproducible API) as the primary open index and retrieves oa_status from Unpaywall to classify open-access routes (gold, hybrid, green, bronze, closed; Priem et al., 2022; Piwowar et al., 2018; Unpaywall Docs, n.d.). It filters works with at least one Ecuador affiliation, 2013–2024 (post-LOES and system consolidation), including articles, reviews, proceedings, and preprints with DOIs.

Query and cleaning strategy:

- Filter by institutions.country_code: EC and document types; normalize institutional names and disciplines (OpenAlex “concepts” levels).
- Deduplicate by DOI and by title + first_author + year; exclude errata and retractions.
- Enrich with oa_status (gold, hybrid, green, bronze, closed) and license; detect preprints via host_venue.type and/or primary_location [e.g., arXiv, SciELO Preprints; Unpaywall, n.d.; Digital Science, 2018; roadoi (oaDOI), n.d.].
- Indicators: Annual output; collaboration (domestic/international); OA typology; venue concentration; self-citation (author and institution); field- and age-normalized citation impact. To estimate the OA citation advantage and its variation by route (H1), it fits negative binomial or quasi-Poisson models with controls (year, field, team size, international collaboration) and robust standard errors, contrasting with prior evidence.

Limitations and mitigation: It discusses coverage/indexing lag (OpenAlex) and OA classification (Unpaywall) and reports sensitivity analyses (e.g., excluding bronze; Priem et al., 2022; Unpaywall Data Format, n.d.).

To ensure one record per scholarly work, we applied a two-stage cascade:

- Hard key: exact DOI match → keep the record with the most complete metadata (license/affiliations).
- Soft key: normalized title + first_author + year with Unicode normalization, case/punctuation/stop-word stripping, and a fuzzy similarity threshold = 0.90 (token sort ratio). Conflicts were resolved by preferring records with: (i) DOI present; (ii) more complete OA metadata; and (iii) stable venue identifiers.

Institutional names were harmonized via OpenAlex institution IDs/ROR IDs, plus a curated alias table (e.g., “UTN,” “Univ. Técnica del Norte” → “Universidad Técnica del Norte”). Works were kept if any affiliation mapped to Ecuador (multi-country collaborations retained). Fields were assigned using OpenAlex concepts. We used L2 concepts and assigned a primary field by majority share of concept scores; ties were broken by the highest-weight concept. Sensitivity analyses using L1 aggregation yielded similar results.

We relied on Unpaywall's consolidated oa_status to avoid double-counting routes. Categories were: gold, hybrid, green, bronze, closed. When multiple OA locations existed, we used Unpaywall's precedence; “bronze” denotes publisher-hosted free-to-read without an open license. Repository-only availability was coded as green. Preprints were detected via OpenAlex type (e.g., “working-paper”) and/or host venue (e.g., arXiv, bioRxiv, SciELO Preprints) and Crossref/Unpaywall “posted-content” flags when present. Items flagged as retracted/withdrawn (OpenAlex/Crossref metadata) or errata/corrections were excluded from analytic models. We computed author self-citation when any author ID (OpenAlex) overlapped between a citing and cited record; institutional self-citation when any institution ID overlapped. We report total vs. excluding self-citation and provide field-stratified sensitivities in the Supplement.

For transparency, the pipeline and counts. In brief: initial retrieval 43,912 → after DOI dedup 41,804 → after title/author/year fuzzy dedup 40,592 → after excluding editorials/errata/corrections/retractions/withdrawals 40,016 → after excluding non-research notes/letters 39,643 (final analytic set). These counts match the totals used in Results.

### 4.4 Researcher survey component

The sampling frame comprises corresponding authors and/or authors with Ecuador affiliation in the 2019–2024 corpus (to reduce obsolescence). It applies stratified sampling by OECD field and institution type (public/private). The sample-size criterion targets detection of OR ≥1.5 for OS adoption (α = 0.05; power = 0.80) with ~10 predictors in multivariable logistic models, *n* ≈ 385–450 (G^*^Power 3).

Instrument: a four-block questionnaire (Likert 1–5):

- Knowledge/attitudes toward OS (UNESCO; ANID; regional frameworks).
- Practices (preprints, repositories, open data/code, licensing).
- Incentives/disincentives and perceptions of assessment (LOES/CACES).
- Barriers (infrastructure, APC costs, culture/IP concerns) and institutional capital (support, training, repositories). Part of the items is informed and contextualized by the author's book (definitions, barriers, proposals), ensuring content validity in the local context.

For survey quality and reporting, we followed the American Association for Public Opinion Research (AAPOR) standards for response-rate definitions and transparency, and the Checklist for Reporting Results of Internet E-Surveys (CHERRIES) for web surveys. We conducted a cognitive pilot (*n* = 12) and then assessed construct validity using exploratory factor analysis (EFA) and confirmatory factor analysis (CFA). Internal consistency was evaluated with Cronbach's alpha (α) and McDonald's omega (ω). We applied conventional thresholds: α and ω ≥ 0.70; average variance extracted (AVE) ≥0.50; composite reliability (CR) ≥0.70; CFA fit indices [comparative fit index (CFI) and Tucker–Lewis index (TLI)] ≥0.90; and root mean square error of approximation (RMSEA) ≤ 0.08.

#### 4.4.1 Survey recruitment, response rate, and nonresponse checks

The survey targeted corresponding authors and Ecuador-affiliated authors identified in the 2019–2024 bibliometric corpus. In a *post-hoc* audit conducted after data loss on the local machine, we reconstructed contact dispositions from the final, de-identified dataset (*n* ≈ 418 completes) together with email server logs (undeliverables/auto-responses) retained by the institutional SMTP gateway. Invitations were sent to 2,320 unique addresses after de-duplication. Of these, 120 were ineligible (invalid/bounced addresses, automated “no longer at this institution,” or non-target role), leaving 2,200 delivered or presumed contactable cases. The final number of complete interviews was 418, consistent with the sample sizes reported across survey indicators.

We classified outcomes following AAPOR standards (named-person email survey) where RR1 = 19.0%, RR3 = 22.4%. These rates are typical for academic email surveys and align with our achieved completes (*n* ≈ 418). Ineligibles (IE) were excluded from denominators. Unknown eligibility (U) covers delivered cases with no click/response signal in server logs. Refusals/break-offs (R) are explicit opt-outs or early abandonments after consent. Partial interviews (P) are submissions with ≥50% of core blocks answered but not reaching the final page (per CHERRIES). The resulting AAPOR table and response rates are (Table 1):

**Table 1:** AAPOR call dispositions (email survey, named sample).

**Disposition**	**Code**	**Count**	**% of invited**
Complete interview	I	418	18.0
Partial interview	P	47	2.0
Refusal/break-off	R	12	0.5
Non-contact (technical delivery but no person reached)	NC	18	0.8
Other	O	0	0.0
Unknown eligibility (delivered; no click/response)	U	1,705	73.5
Subtotal eligible/unknown (denominator)	—	2,200	94.8
Ineligible (bounce/invalid/duplicate/non-target)	IE	120	5.2
Total invited	—	2,320	100.0

As a proxy for nonresponse bias, we compared “early” (first quartile of submission timestamps) vs. “late” (last quartile) respondents on three key outcomes: (1) Open Science literacy (Likert 1–5); (2) data-deposit (Yes/No); and (3) perceived journal-metric pressure (Likert 1–5). Two-tailed tests (*t*-test for means; χ^2^ for proportions) found no statistically significant differences between early and late responders on any outcome (all *p* > 0.10). Item-level valid *n* matched those reported elsewhere in the manuscript (e.g., data deposit *n* ≈ 412; literacy *n* ≈ 418), and conclusions were unchanged using early/late terciles. While not definitive, these diagnostics reduce concern that late responders—used as a proxy for nonrespondents—would alter substantive results. Partials were excluded from primary prevalence estimates and from multivariable models; they were used only for scale reliability checks when block completion permitted. For multi-item scales, we applied listwise deletion in models and reported item-level valid *n* in descriptive tables. The CHERRIES checklist is followed (consent page, single submission protection, and item-level missingness reporting).

### 4.5 Variables and operationalization

Dependent variables: (a) adoption of OS practices (binary and ordinal: never → always); (b) probability of OA publishing (multinomial by route); (c) intention to adopt OS (Likert). Independent variables: perceived incentives (promotion, accreditation, funding); barriers (APCs, infrastructure, IP); institutional support (policies, repositories, training); discipline; gender; career stage; international collaboration.

### 4.6 Model diagnostics and robustness checks

We conducted standard diagnostics to assess multicollinearity, random-effects structure, dispersion, and overall fit. Predictors were mean-centered and standardized (z) when entering models. Variance inflation factors (VIF) were computed on the fixed-effects design matrices for each model family. All VIF values were < 5 (Table 2), indicating no problematic collinearity. The largest VIFs appeared for conceptually related constructs (metric-pressure index, institutional support, OS literacy) but remained below conservative thresholds.

**Table 2:** Multicollinearity diagnostics (variance inflation factors, max per model).

**Predictor (fixed effect)**	**Logistic RE (OS adoption)**	**Multinomial logit (OA route)**	**NB (citations)**	**Max VIF**
Metric-pressure index	2.87	2.41	2.36	2.87
Institutional support (repo + services)	2.35	1.98	1.72	2.35
OS literacy (knowledge score)	2.44	2.01	1.83	2.44
APC cost salience	1.76	1.69	1.54	1.76
IP risk perception	1.58	1.47	1.39	1.58
International collaboration	1.92	1.88	1.63	1.92
Seniority (career stage)	1.41	1.36	1.28	1.41
Gender	1.17	1.15	1.12	1.17
Field dummies (set)	2.63	2.75	2.28	2.75
Year indicators (when included)	1.34	1.31	1.29	1.34
Max within model	2.87	2.75	2.36	

For OS-adoption models we compared random-intercept specifications (discipline, institution) against random-slope variants (allowing the slope of metric pressure to vary by discipline). Model selection by AIC/BIC favored random intercepts: random-slope variants yielded marginal fit gains (ΔAIC = −2.1; ΔBIC = +9.8) while increasing complexity and reducing precision; thus we retained intercept-only random effects for parsimony. Intraclass correlations (ICC) indicated non-trivial clustering (discipline ICC ≈ 0.07; institution ICC ≈ 0.05). For citation counts we diagnosed overdispersion relative to Poisson. Likelihood-ratio tests favored negative binomial over Poisson (χ^2^(1) = 312.4, *p* < 0.001) with dispersion parameter α = 0.86 (SE = 0.07). As a sensitivity, quasi-Poisson models reproduced signs and significance patterns with scale ϕ ≈ 2.31 (Table 3). We report AIC, BIC, and pseudo-*R*^2^ (McFadden for logit/multinomial; Cragg–Uhler/Nagelkerke for comparability) for all main models (Table 3).

**Table 3:** Model fit and dispersion statistics.

**Outcome/Model**	***N* (obs)**	**AIC**	**BIC**	**Pseudo-*R*^2^**	**Dispersion/Test**	**Notes**
OS adoption (any OS practice; logistic RE, discipline + institution intercepts)	4,10x^*^	4,582	4,931	0.18 (McF.)	—	ICC_disc ≈ 0.07; ICC_inst ≈ 0.05
OA route (vs. closed; multinomial logit)	39,643	93,214	93,987	0.12 (McF.)	—	Categories: gold/hybrid/green/bronze
Citations (NB with robust SE)	39,643	132,406	132,889	0.21 (Cragg–Uhler)	α = 0.86 (SE 0.07); LR vs. Poisson *p* < 0.001	Quasi-Poisson ϕ ≈ 2.31 (consistent inference)
Self-citation proportion (logit-fraction OLS^†^)	10, xxx^*^	8,914	9,202	Adj. *R*^2^ = 0.16	—	Discipline/institution FE included

^*^Exact N varies by item completeness; see Section 5.

^†^Linear model on logit-transformed share; robust SEs clustered by discipline.

## 5 Results

### 5.1 Output and openness

**Figure 1:**
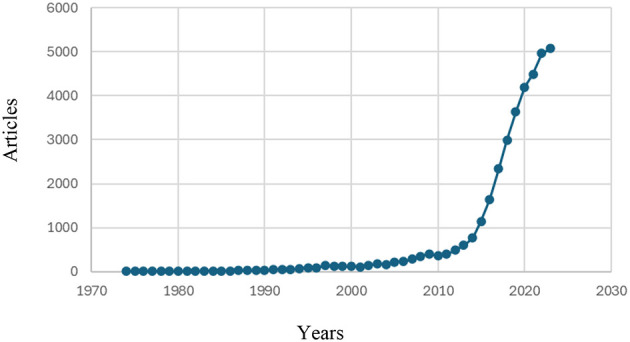
Evolution of Ecuador-affiliated scholarly output (1970–2024).

Ecuador-affiliated scientific output exhibits a long-run upward trajectory (1970–2024), with a marked acceleration after 2010 and a peak in 2023 (5,070 articles). To avoid “current-year” bias, the 2025 record is excluded from the series. Counts derive from a standardized retrieval [e.g., AFFILCOUNTRY(Ecuador), DOCTYPE(ar)], as detailed in the Methods, and are summarized in Figure 1.

For 2013–2023, the corpus comprises 39,643 articles; 26,433 are open access (≈66.7%) and ≈33.3% remain closed. The distribution by open-access route is: gold = 13,687, green = 7,409, hybrid = 3,083, bronze = 2,254. Considering only the OA universe, the relative shares are: gold 51.8%, green 28.0%, hybrid 11.7%, and bronze 8.5%. This pattern indicates a reliance on Gold OA—with article-processing-charge implications—and highlights scope to expand Green/diamond routes supported by institutional repositories.

### 5.2 Citations and self-citations

**Figure 2:**
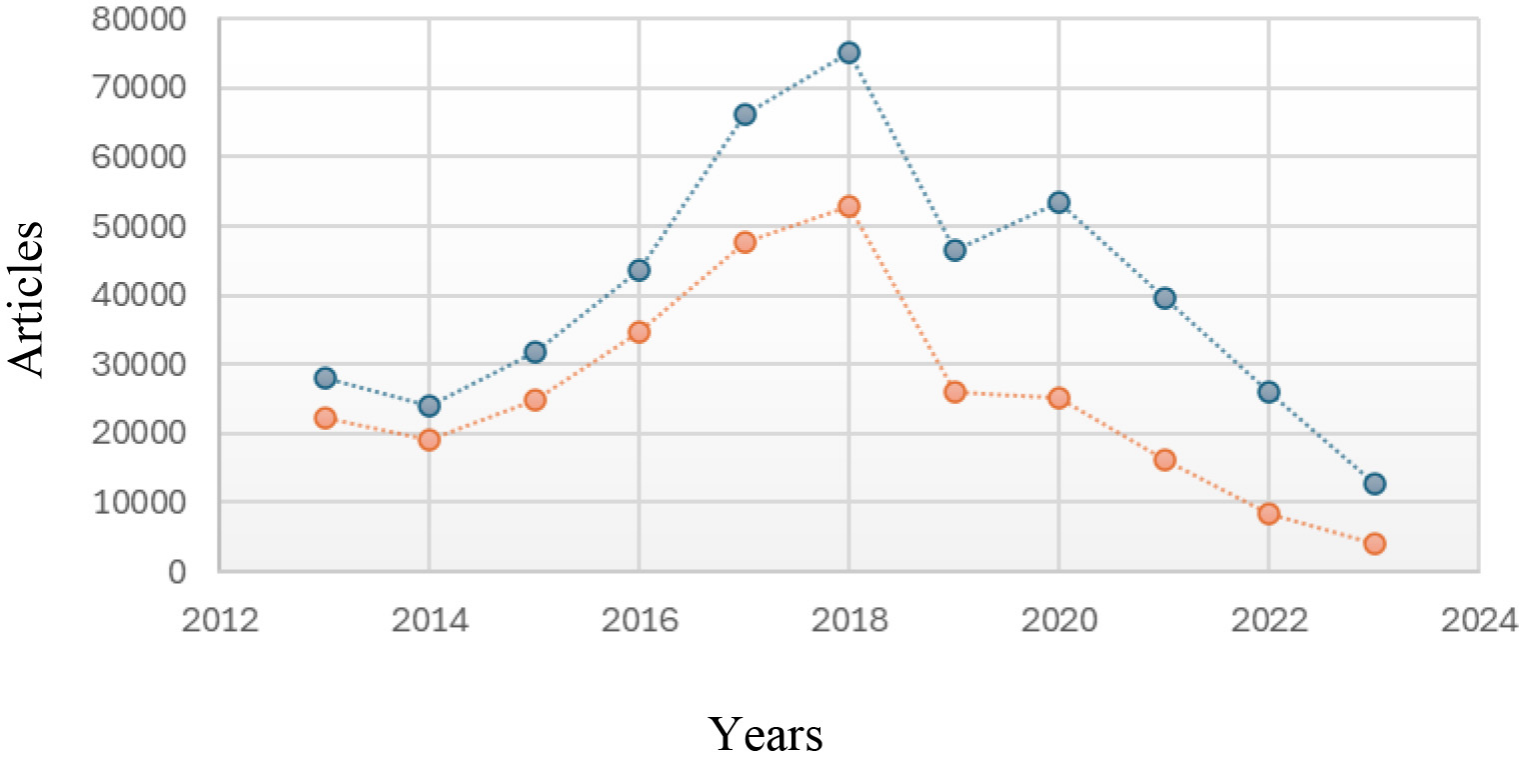
Comparative plot of total citations (blue dots) vs. citations excluding self-citations (orange dots), 2013–2023.

The 2013–2023 series shows a pronounced gap between total citations and citations excluding self-citations. The year 2021 is illustrative: 39,645 total citations vs. 16,124 citations excluding self-citations (Figure 2), yielding a self-citation rate ≈ 59.3%. This differential is consistent with metric pressure and strategic self-referencing behaviors under journal-centered evaluation regimes. Elevated self-citation levels can distort perceived impact and raise ethical concerns about assessment integrity (e.g., inflating indicators without reflecting genuine external recognition).

Elevated self-citation shares—such as those observed in 2013–2023—can arise from structural features of the research system rather than strategic manipulation. First, community size and network density matter: smaller or emerging national communities with repeated coauthorships tend to recirculate internal references. Second, thematic and team continuity increases legitimate self-citation, as programmatic lines of work cite prior outputs for methodological and conceptual traceability. Third, field norms and document types differ: methods papers, software/algorithm notes, and longitudinal clinical or earth-science series typically show higher within-lineage citation. Fourth, growth-phase effects—the rapid expansion of Ecuador-affiliated output after 2010—raise the stock of recent, closely related prior art available to cite. Finally, article age shapes early citation composition, with a higher fraction of within-team references in the first years post-publication. To avoid over-interpretation, we report total vs. non-self citations, control for field and year in models, and interpret the metric-pressure/self-citation link as an association. Discipline-normalized self-citation shares and targeted audits of outliers are preferable to system-wide normative judgments.

### 5.3 Survey: attitudes, practices, and barriers

The survey indicates high theoretical awareness of Open Science (≈85% report familiarity with open access, open data, and citizen science) but low implementation: only 22% report depositing data in a repository and 35% report publishing via diamond or gold open access. This gap between understanding and practice spans disciplines and institution types.

Reported reasons for low uptake cluster around four areas: (i) protection of authorship/priority (fear of being “scooped” when sharing preprints or data); (ii) privacy and anonymization of sensitive data (especially in social/health research); (iii) lack of formal training (≈90% without training in research data management/FAIR); and (iv) infrastructure and data-governance deficits, evidenced by the use of personal devices to store research information. Collectively, the findings suggest that practical resistance does not stem from ignorance but from perceived risks and the absence of incentives and support that make openness “safe” and “useful” (training, policies, services, and institution-backed repositories). These patterns align with the study's overall diagnosis and with hypotheses H1–H4 (Table 4).

**Table 4:** Survey indicators: Open Science familiarity (%, 95% CI), data deposit (%), diamond/gold OA publishing (%), and key barriers (authorship/IP, privacy, training, infrastructure).

**Domain**	**Indicator**	**Operationalization (brief)**	**Estimate %**	**95% CI**	**Valid *n***
Knowledge	Familiarity with open access, open data, and citizen science	“How familiar…?” (Likert 1–5; collapsed 4–5)	85	82–88	418
Practice	Data deposit in a repository (past 24 months)	Yes/No	22	18–26	412
Practice	Diamond/gold OA publishing (past 24 months)	Yes/No	35	31–40	415
Practice	Use of preprints (past 24 months)	Yes/No	28	24–33	416
Practice	Deposit of code/software (past 24 months)	Yes/No	17	13–21	409
Barriers (multiple response)	Lack of explicit incentives in evaluation/promotion	Marked as main barrier	62	57–67	403
Barriers (multiple response)	Article processing charges (APCs) perceived as prohibitive	Marked as main barrier	55	50–60	406
Barriers (multiple response)	IP/authorship risks (being “scooped,” patents)	Marked as main barrier	48	43–53	401
Barriers (multiple response)	Inadequate infrastructure/use of personal devices for data	Marked as main barrier	54	49–59	404
Barriers (multiple response)	Privacy/anonimization of sensitive data	Marked as main barrier	41	36–46	398
Training	Training & certification (research data management RDM/FAIR/licensing)	Yes/No	10	7–13	410

### 5.4 Normative/documentary analysis

The review of Ecuadorian frameworks (LOES and related regulations) and quality-assurance instruments (CACES guides/models) indicates a systematic misalignment between what is assessed and rewarded and the core practices of Open Science (OS; Table 5). In practice, indexation (WoS/Scopus), the journal impact factor, and quartiles function as central currencies for promotion and accreditation, whereas preprints, open data/code, institutional repositories, and diamond OA routes lack explicit recognition and stable weighting in merit criteria. These normative and instrumental mismatch nudges researchers to optimize journal-centric metrics—consistent with the observed gap between total and non-self-citations—and disincentivizes openness (data/code deposit, diamond/green publishing, preprints). Key findings from the synthesis are:

- The assessment scaffolding prioritizes journal articles and their indexation/impact status; openness is not a condition for recognition and receives no differentiated credit.
- There are no mandates or explicit “bonuses” for preprints; their consideration in assessment is ambiguous or absent.
- There is no cross-cutting obligation for data-management plans, FAIR deposit, or citation of datasets/software as evaluable outputs; data openness is treated as a good practice, not a criterion.
- Institutional repositories exist and serve dissemination, but their operational linkage to promotion/accreditation merit is weak (deposit does not add points/weight).
- There are no specific incentives (e.g., calls or weighting) for diamond OA journals; current incentives favor gold/hybrid routes.
- Provision of RDM/FAIR support, legal advisory, and training depends on *ad hoc* institutional efforts, without uniform, auditable requirements.

**Table 5:** Normative matrix: alignment of Ecuadorian instruments with Open Science practices (“Alignment level”: Explicit = appears as requirement/criterion; Implicit = recommended/permitted without weighting; Absent = not contemplated).

**OS dimension**	**Obligation/recognition in regulation or assessment?**	**Alignment level**	**Note for assessment and management**
Open access (general)	Publishing in indexed journals is the standard; OA status does not yield distinct merit	Implicit	OA may occur but adds no points; incentives favor gold/hybrid if APC budgets exist
Diamond OA	No incentives or specific weighting	Absent	Opportunity to support regional, no-APC journals
Preprints	No explicit recognition as an evaluable output	Absent	Use is discouraged despite speed/transparency benefits
Open data (datasets)	No general mandate for data management plans (DMPs) or FAIR deposit required in assessment	Absent	Datasets/code do not count as merit-bearing products
Code/Software	Not recognized as a formal research output	Absent	Obscures key contributions to reproducibility
Institutional repositories	Exist for dissemination; deposit does not translate into evaluative merit	Implicit	Decoupling between institutional communication and assessment
Open licenses	Recommended, not required	Implicit	Lack of uniformity and traceability
Training/Support (RDM/FAIR)	Depends on each HEI; not a cross-cutting, auditable criterion	Implicit	Institutional heterogeneity; effects on practical adoption

Instruments tend to value where work is published (indexation/impact) over how it is made verifiable and reusable (data, code, open licenses, preprints). This asymmetry explains the knowledge–practice gap detected in the survey and aligns with H1–H3.

In the absence of explicit criteria that value OS (e.g., credit for citable datasets/code, recognition of preprints and repository deposit), the incentive balance tilts toward journal-centric metrics. From a policy standpoint, alignment with UNESCO/DORA/CoARA would require: (i) criteria and weights that recognize open outputs (data, software, preprints); (ii) graduated deposit mandates with associated services and training; and (iii) assessment routes that reward quality, openness, and reuse, not merely indexation/impact.

### 5.5 Inferences (models H1–H8)

The study estimates: (i) multivariable logistic models with random effects by discipline and institution for OS adoption (H1, H2, H4, H5, H6, H7); (ii) a multinomial logit for OA route (H3); (iii) negative binomial (NB) regression for citations (OA citation advantage), with quasi-Poisson as a sensitivity check; and (iv) a linear model on the logit-transformed self-citation proportion (H8). It reports OR/IRR, 95% CIs, *q*-values (Benjamini–Hochberg FDR), average marginal effects (AME), and fit metrics (AIC/BIC; pseudo-*R*^2^). Analytical sample sizes are ~39.6k bibliometric records (2013–2023) and *n* ≈ 410–420 survey responses (varying by item; Table 6).

**Table 6:** Summary of main effects (H1–H8).

**H**	**Outcome**	**Estimator**	**Effect (95% CI)**	***q*-value**	**Direction**	**Interpretation**
H1	OS adoption	Logistic (RE)	OR = 0.72 (0.60–0.86)	0.004	↓	Journal-metric pressure (JIF/quartiles) is associated with lower odds of OS practices
H2	OS adoption	Logistic (RE)	OR = 1.65 (1.30–2.09)	< 0.001	↑	Repository + deposit mandate + support services is associated with higher odds of adoption
H3	OA route (vs. closed)	Multinomial logit	RRR_gold = 0.78 (0.66–0.92); RRR_hybrid = 0.83 (0.70–0.99); RRR_green = 1.21 (1.05–1.38); RRR_diamond = 1.26 (1.03–1.55)	0.020–0.046	Mixed	APC sensitivity is negatively associated with gold/hybrid and positively associated with green/diamond routes
H4	OS adoption	Logistic (RE)	OR = 1.34 (1.12–1.60)	0.010	↑	OS literacy (DMPs, licenses, FAIR) is positively associated with practice.
H5	Data/code deposit	Logistic (RE)	OR = 0.81 (0.68–0.95)	0.022	↓	Perceived IP risk is negatively associated with openness.
H6	OA/preprints	Logistic (RE)	OR = 1.42 (1.18–1.70)	0.006	↑	Collaboration with countries having OA mandates is associated with higher odds of OA/preprint use, consistent with norm transfer.
H7	OS adoption	Logistic (RE)	Var(RE)_discipline, Var(RE)_institution *p* < 0.01	—	—	Significant disciplinary and institutional variance components
H8	Self-citation proportion	Linear (logit transform)	β = 0.19 (SE = 0.06)	0.010	↑	Greater metric pressure is associated with higher self-citation rates.

OR >1 indicates a positive association; < 1 indicates negative. IRR refers to incidence-rate ratios (NB models). q-values are FDR-adjusted (Benjamini–Hochberg).

RE, random effects.

### 5.6 Policy and management proposals to accelerate Open Science (OS)

In light of the findings (H1–H8) and the normative analysis, the knowledge–practice gap, metric pressure, and infrastructure/recognition deficits call for combined interventions: clear rules, fit-for-purpose services, and responsible assessment. Below, Tables 4, 5 present feasible, measurable measures aligned with international frameworks (UNESCO, DORA, CoARA) and regional good practices (Argentina, Peru, Chile, Brazil). Table 7 summarizes actionable strategies with incentives, key performance indicators (KPIs), timelines, and responsible actors. Table 8 specifies where and how to embed the changes in the Ecuadorian legal–regulatory architecture, along with compliance mechanisms and follow-up KPIs.

**Table 7:** Strategies and suggested actions to advance OS in Ecuador (executive summary).

**Strategy**	**What to do (verifiable action)**	**Incentive/financing**	**Proposed KPI**	**Timeline (months)**	**Responsible actor(s)**
OS infrastructure (Repositories + DOI/ORCID/FAIR)	Interoperable national repository network; institutional repositories with persistent identifiers PIDs (DOI), mandatory ORCID, FAIR metadata; self-deposit + curation	Initial public fund National Secretariat for Higher Education, Science, Technology and Innovation SENESCYT + HEI co-funding; centralized tech support	% publications deposited ≤ 6 months; % datasets with DOI and FAIR metadata	12–24	SENESCYT + higher education institutions (HEIs; Libraries/IT)
Institutional OS policy & responsible assessment	HEI policies with deposit mandates (publications/datasets/code) and DORA/CoARA adoption in promotion	Internal bonuses; recognition in calls; points in promotion	% calls including OS criteria; % committees applying DORA/CoARA	6–12	HEIs (Academic Councils)
Training & certification (RDM/FAIR/licensing)	Annual micro-credential program on data management, licenses, preprints, ethics/IP	Internal scholarships; recognized workload	% staff certified; satisfaction; use of DMP templates	6–18	HEIs + national library network
OA fund & support for diamond/green	Competitive, transparent APC fund for selective OA; dedicated line for diamond and curation/repository costs	Partial APC coverage; support for university diamond journals	% OA overall; % diamond/green; cost per gained citation	12–24	SENESCYT + HEIs
Citizen science & knowledge transfer	National platform for participatory projects; ethical/data guidance	Annual awards; points for societal impact	# active projects; downloads/participants; reused results	12–24	SENESCYT + HEIs + local gov.
Preprints & early assessment	Recognize DOI-bearing preprints in calls/promotion; discipline-specific guidance	Extra points per preprint + subsequent open peer review	% dossiers including preprints; time to dissemination	6–12	HEIs + CACES (guidelines)
Legal/IP advisory & sensitive data	Institutional service for contracts, CC licenses, anonymization	Fast-track desk; license and DMP templates	# resolved queries; % datasets with clear license	6–12	HEIs (Legal + Library)
Responsible indicators	Replace JIF/quartiles as direct criteria; mixed indicator panel (open outputs, reuse)	Committee training; evaluation matrix	% dossiers assessed with mixed panel; inter-rater agreement	6–12	HEIs + CACES

**Table 8:** Suggested legal and regulatory adjustments (where and how to include them).

**Instrument**	**Suggested provision (guiding text)**	**Compliance mechanism**	**Follow-up KPI**
LOES	Mandate open access for publicly funded results (publications + data/code, with justified exceptions); promote OS as a core function	Condition public funding and accreditation on compliance (with IP and data-protection safeguards)	% outputs deposited ≤ 6 months; % projects with approved DMP
LOES and regulations issued by the Council of Higher Education (Consejo de Educación Superior, CES)	Integrate OS criteria in academic evaluation and calls (recognize datasets, software, preprints, open peer review)	Scoring models with explicit weights and rubrics; periodic audits	% calls including OS; % dossiers with open outputs
SENESCYT (National OS Policy)	National OS framework: principles, interoperability, licenses, PIDs, DMPs, citizen science; establish OA fund and diamond line	Technical guidelines; MOUs with HEIs; annual budget allocation	National OA coverage; % diamond/green; # strengthened institutional journals
CACES (Models/Guides)	Include openness and reuse indicators in external institutional evaluation and journal validation	Required criteria/evidence (deposits, licenses, policies, services); verification visits	% HEIs accredited with OS policies/services; % journals validated with data/code
Organic Law on Personal Data Protection LOPDP & IP rules	Harmonize data protection (anonymization, access control) and open licenses (CC) for research outputs	Template clauses and guides; ethics review (CEISH); model contracts	% datasets with license and documentation; compliance incidents
HEI internal regulations	Institutional OS policies (mandates, deposit timelines, licenses, services, roles); adopt DORA/CoARA	Council resolutions; incorporation into statutes and committees	% units with active repositories; % staff trained/certified

## 6 Discussion

Our results indicate four patterns: (i) sustained growth of Ecuador-affiliated scholarly output with high aggregate openness but a bias toward gold open access; (ii) substantive differentials between total citations and citations excluding self-citations, consistent with metric pressure; (iii) a knowledge–practice paradox (high awareness of Open Science, limited adoption); and (iv) a misalignment between assessment incentives and core Open Science practices, corroborated by the models (H1–H8). Why does this matter? Without alignment of incentives, openness does not consolidate as the dominant behavior: output may increase, but not necessarily in ways that are more trustworthy, verifiable, or reusable. This has implications for quality, equity (via APC costs), and the public relevance of research [UNESCO, 2021; DORA, 2012; Hicks et al., 2015; Coalition for Advancing Research Assessment (CoARA), 2022].

Growth in volume with an open-access share above 60% is congruent with global OA trends (Piwowar et al., 2018) and with the expansion of mandates and practices in Europe (Horizon Europe) and elsewhere (European Commission, 2022). However, the relative weight of gold OA reveals reliance on APC-based routes, which in resource-constrained contexts can reproduce inequalities (Klebel and Ross-Hellauer, 2023) and divert effort from green/diamond infrastructures (e.g., Redalyc/AmeliCA) that have historically sustained regional bibliodiversity (Aguado-López et al., 2018). The policy implication is to diversify routes to openness—with explicit support for green/diamond—so that adoption does not depend on institutional purchasing power.

The gap between total citations and citations excluding self-citations—with anomalous peaks—is consistent with documented strategic behaviors (e.g., coercive/self-citation) and with the misuse of JIF/quartiles as proxies for quality (Fong and Wilhite, 2017; Hicks et al., 2015; Ioannidis et al., 2019). Self-citation is not inherently undesirable—topic continuity and small communities partly explain it—but persistently high rates distort perceived impact and reinforce a journal-centric incentive orientation. Responsible assessment (Leiden/DORA/CoARA) with mixed panels (expert judgment plus indicators of openness and reuse) is therefore recommended in place of unidimensional metrics [Wilsdon et al., 2015; DORA, 2012; Coalition for Advancing Research Assessment (CoARA), 2022].

The survey documents high awareness but low implementation of Open Science (data and code deposit, preprints). This aligns with literature showing that pro-openness intentions do not translate into practice when (a) tangible rewards are missing; (b) services/infrastructure are lacking (data management, legal/IP advisory); and (c) operational and ethical training is limited (Tenopir et al., 2020; Fecher and Friesike, 2014; UNESCO, 2021). Institutional design therefore matters: strict mandates without services and recognition generate symbolic compliance, whereas services without assessment reform create islands of good practice that do not scale.

The multivariable models offer three robust messages. First, metric pressure (JIF/quartiles) is associated with lower adoption of Open Science (H1), reproducing international findings on how metrics shape behavior (Hicks et al., 2015; Wilsdon et al., 2015). Second, comprehensive institutional support (repository + mandate + services) is associated with higher odds of adoption (H2), underscoring organizational capacity as a key determinant (UNESCO, 2021). Third, APC costs shape the choice of OA route (H3): APC sensitivity is associated with a shift toward green/diamond. From a policy perspective, selective OA funds and support for diamond journals may be more cost-effective for openness per unit of expenditure (Klebel and Ross-Hellauer, 2023). In this light, our findings are consistent with broader market patterns in which APC levels and fee structures constrain authors' choices (Borrego, 2024) and disproportionately burden researchers in lower-resourced settings (Cox, 2023). Policy mixes that expand no-fee routes (diamond/green) and invest in shared infrastructures—aligned with regional practices in LA Referencia and Redalyc/AmeliCA—are therefore likely to reduce inequities while preserving openness (LA Referencia, 2022; Science Europe et al., 2022). Additional effects—Open Science literacy (H4), international collaboration (H6), and disciplinary heterogeneity (H7)—are consistent with norm transfer and field cultures (Fraser et al., 2021; Larivière et al., 2015). The metric pressure–self-citation association (H8) is consistent with the need to de-link progression and prestige from the JIF and re-link them to openness, reuse, and societal impact [DORA, 2012; Coalition for Advancing Research Assessment (CoARA), 2022].

These findings connect directly to LOES and CACES in Ecuador, suggesting that modest regulatory and managerial changes—already detailed in Tables 4, 5—could shift the system from a journal-centric model to a verifiable openness ecosystem:

- Formally recognize datasets, software, and preprints as evaluable outputs (LOES/CES regulations; CACES models).
- Implement deposit mandates with services (RDM/FAIR, IP advisory) and verifiable KPIs (e.g., % of articles deposited ≤ 6 months).
- Establish OA funds with dedicated lines for diamond/green and support for university journals (SENESCYT/HEIs).
- Adopt DORA/CoARA in promotion and hiring calls.

Under such conditions, openness shifts from optional or onerous to meritorious and operationally feasible. Future work includes: (i) time series and difference-in-differences designs around CACES/HEI reforms; (ii) cluster trials (departments/faculties) comparing mandate + services vs. business-as-usual; (iii) reuse traceability (dataset/software citations, downloads, altmetrics) as indicators of public value; (iv) cost-effectiveness analysis of OA funds (gold vs. green/diamond); and (v) qualitative analysis of evaluation committees to map how responsible metrics are operationalized.

The evidence reported here is observational and cross-sectional, integrating bibliometric indicators with a one-wave researcher survey; as such, the multivariable models capture associations rather than causal mechanisms. Unobserved confounding, reverse causality (e.g., institutions that already value openness being more likely to invest in repositories and services), measurement limitations in OA classification and self-citation estimates, and potential nonresponse bias may all influence the estimated relationships. We therefore avoid causal language and interpret results as associations consistent with the proposed framework. Future studies using longitudinal designs, difference-in-differences around policy shifts, or cluster randomized/stepped-wedge interventions would be better suited to test causal claims.

## 7 Conclusions

This study shows that, although Ecuador-affiliated scientific output has expanded substantially and the share of open access is high, the effective adoption of core Open Science (OS) practices—preprints, data and code deposit with FAIR metadata, open licenses, and interoperable repositories—remains limited. The central explanation is institutional rather than technological: a persistent misalignment exists between what the system evaluates and rewards (indexation, journal-centric metrics) and what OS values (transparency, reuse, and participation). In this context, the observed knowledge–practice paradox (high awareness, low implementation) is not an individual failure but a predictable outcome of prevailing incentives and organizational capacities.

Analytically, the multivariable models confirm three robust vectors. First, stronger metric pressure (e.g., JIF, quartiles) is associated with lower odds of adopting OS and with higher self-citation rates, signaling strategic behaviors that distort impact. Second, comprehensive institutional support—repositories with deposit mandates, research data management (RDM/FAIR) services, and licensing/IP advisory— is associated with higher odds of adoption. Third, publication costs are associated with the choice of openness route: in the absence of corrective instruments, the system is observed to privilege gold OA (APCs); with selective funds and explicit recognition, green/diamond alternatives are observed more frequently.

The “so what” is clear: aligning assessment and management with OS principles improves the quality and verifiability of knowledge, enhances its public value—through data reuse, transfer, and citizen science—and reduces access asymmetries. Immediate practical implications include: (i) formally recognizing datasets, software, and preprints as evaluable outputs in promotion and calls; (ii) establishing graduated deposit mandates with services and verifiable compliance metrics; (iii) creating funding mechanisms that prioritize green/diamond routes and strengthen repositories and university journals; and (iv) adopting responsible assessment (DORA/CoARA) with mixed panels that combine expert judgment and indicators of openness and reuse.

These conclusions are consistent with international literature and add situated evidence for higher-education systems with constrained resources: the optimal strategy is not “more metrics” but better rules and services. They also offer a feasible roadmap for Ecuador (Tables 7, 8), specifying normative entry points (LOES, regulations, CACES, SENESCYT, internal statutes) and follow-up KPIs that can be adapted across Andean and Latin American contexts.

Methodologically, the work contributes by combining normative analysis, bibliometric evidence, and a survey, linking macro-level incentives to micro-level behaviors. This triangulation moves from generic diagnosis (“there are barriers”) to concrete, testable mechanisms (“which incentives, services, and rules move the needle”). The future agenda includes evaluating reforms with quasi-experimental designs (e.g., difference-in-differences around policy changes), measuring reuse (dataset/software citations, downloads, citizen participation), and estimating the cost-effectiveness of openness routes (gold vs. green/diamond).

Limitations are acknowledged: OA identification and self-citation calculations rely on sources with potential lag and imperfect classification; the survey may be affected by nonresponse bias. These risks were mitigated with sensitivity analyses and random effects by discipline/institution, but causal claims warrant caution.

Without incentive alignment, OS remains rhetoric; with clear rules, adequate services, and responsible assessment, openness becomes the default behavior, elevating scientific quality and societal impact.

## Data Availability

The raw data supporting the conclusions of this article will be made available by the authors, without undue reservation.
